# Supplementary material for: Increased excitatory to inhibitory synaptic ratio in parietal cortex samples from individuals with Alzheimer’s disease
Source: Nat Commun. 2021 May 10;12:2603. doi: 10.1038/s41467-021-22742-8 (PMC8110554; doi:10.1038/s41467-021-22742-8)
Supplement: Supplementary file 1 — Supplementary Information [file 41467_2021_22742_MOESM1_ESM.pdf]

**Increased Functional Excitatory to Inhibitory Synaptic Ratio in Parietal Cortex of Alzheimer's Disease**

Julie C. Lauterborn, Pietro Scaduto, Conor D. Cox, Anton Schulmann, Gary Lynch, Christine M. Gall, C. Dirk Keene, Agenor Limon.

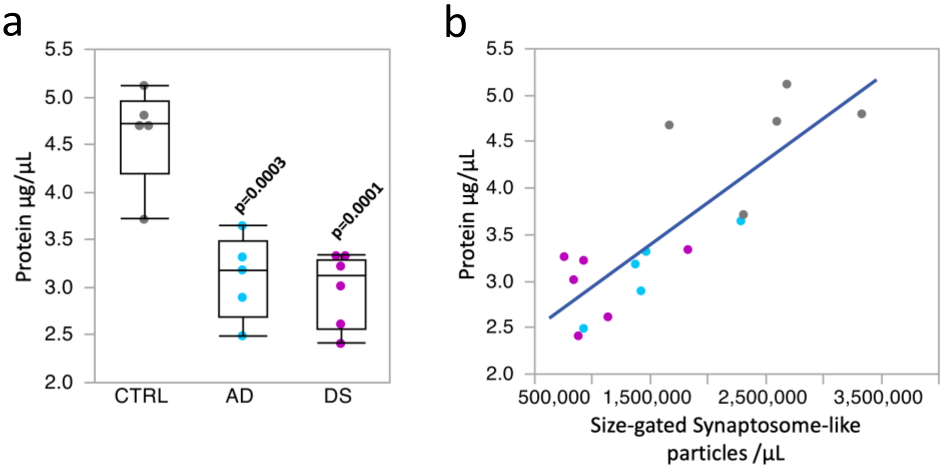


**Supplementary Fig 1. Correlation between numbers of particles within synaptosome size and protein concentration in P2 fractions.** (**a**) Boxplots of the amount of protein in each group. The median is represented by the line within the box, and the first and third quartiles are represented by the ends of the box. The whiskers extend from each end of the box to the first or third quartile ±1.5 (interquartile range). The amount of protein in each group, measured by Quibit, was reduced from 4.6 ± 0.2 µg/µL in control (mean ± SEM; *n* = 5 subjects) to 3.1 ± 0.2 µg/µL in Alzheimer's disease (AD) (*n* = 5 subjects) and 3 ± 0.2 µg/µL in Down Syndrome (DS) (*n* = 6 subjects). Each dot (**a,b**) represents a single subject color coded as control (gray), AD (cyan) and DS (magenta). A one-way analysis of variance showed effects of diagnosis on protein concentration ($F$ (2,13) = 21.5, $P < 0.0001$). Post hoc analysis comparing to control using two-sided Dunnett's method (AD and DS vs CTRL) indicated that the average number of particles was lower in AD and DS. (**b**) The amount of protein was strongly correlated to the number of synaptosomes size gated (1-3 µm in Figure 4) by flow cytometry $R^2$ (16) = 0.68; $P = 8.9$ e$^{-5}$, ($n$ = 5 control, 5AD and 6 DS subjects).

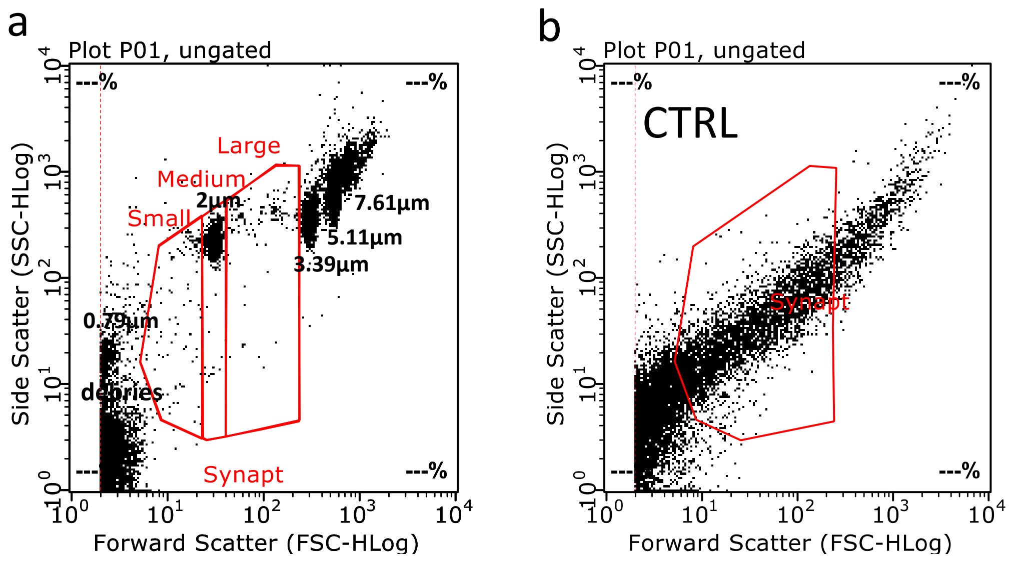


**Supplementary Fig 2. Gating strategy used for size-gating of synaptosome-like particles in P2 preparations.** (**a**) Reference standard size beads (Spherotech, Inc) were first used to exclude particles below 0.79 µm and above 3.39 µm. In addition, the gate was subdivided for analysis in small medium and large size particles. (**b**) The same strategy was used to analyze P2 preparations presented on Fig. 4.

a b

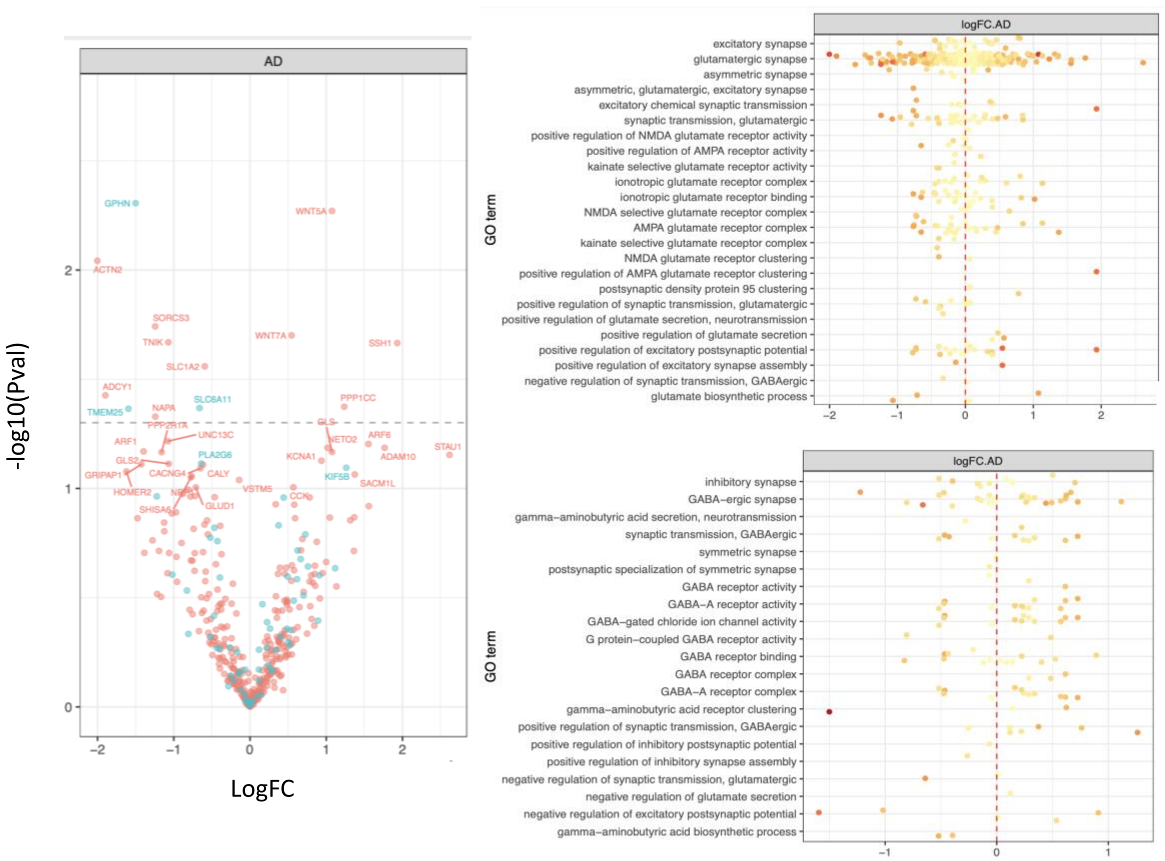


**Supplementary Fig 3. Differential gene expression of predetermined gene ontology (GO) terms.** (**a**) Volcano plot of log2 fold change (logFC) of all excitatory (red) and inhibitory (turquoise) related genes are shown for clinical diagnosis (AD vs. control). Each gene's logFC is a dot. (**b**) Dot plot of excitatory- and inhibitory-related genes by gene ontology category. Each dot corresponds to the logFC value of a gene. Genes are plotted multiple times when they overlap between the categories.

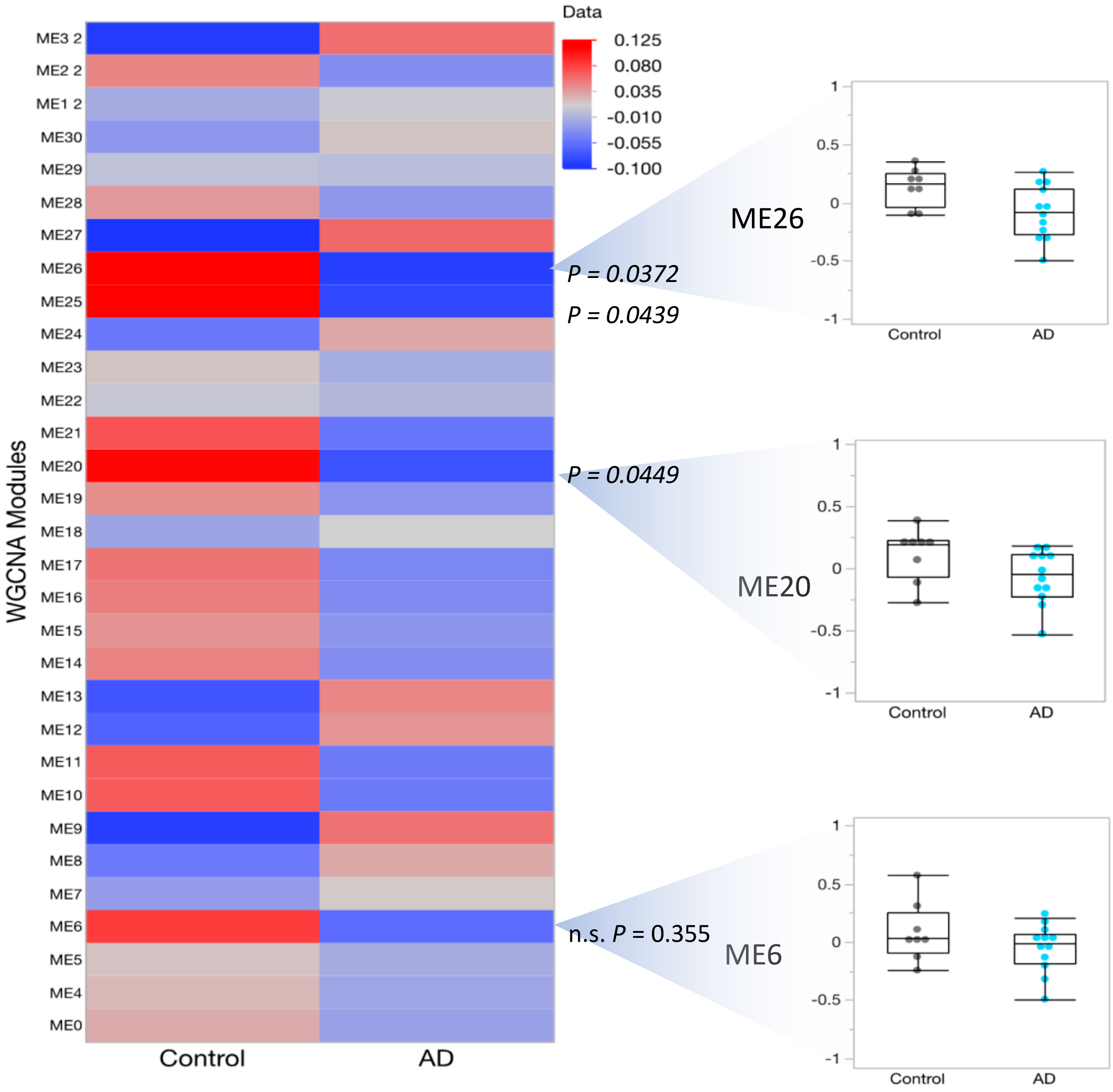


**Supplementary Fig 4. Weighted gene co-expression network analysis (WGCNA) modules in control and AD.** Heat map displaying the average of the eigengene value for 31 modules identified by WGCNA. The *P* values are shown for the three modules that are differentially expressed between control and AD (two-sided Wilcoxon/Mann-Whitney U-test), and one non-significantly different module for comparison. The dots in the box plot indicate the individual eigengene values for the selected modules in each group. The median is represented by the line within the box, and the first and third quartiles are represented by the ends of the box. The whiskers extend from each end of the box to the first or third quartile ±1.5 (interquartile range).

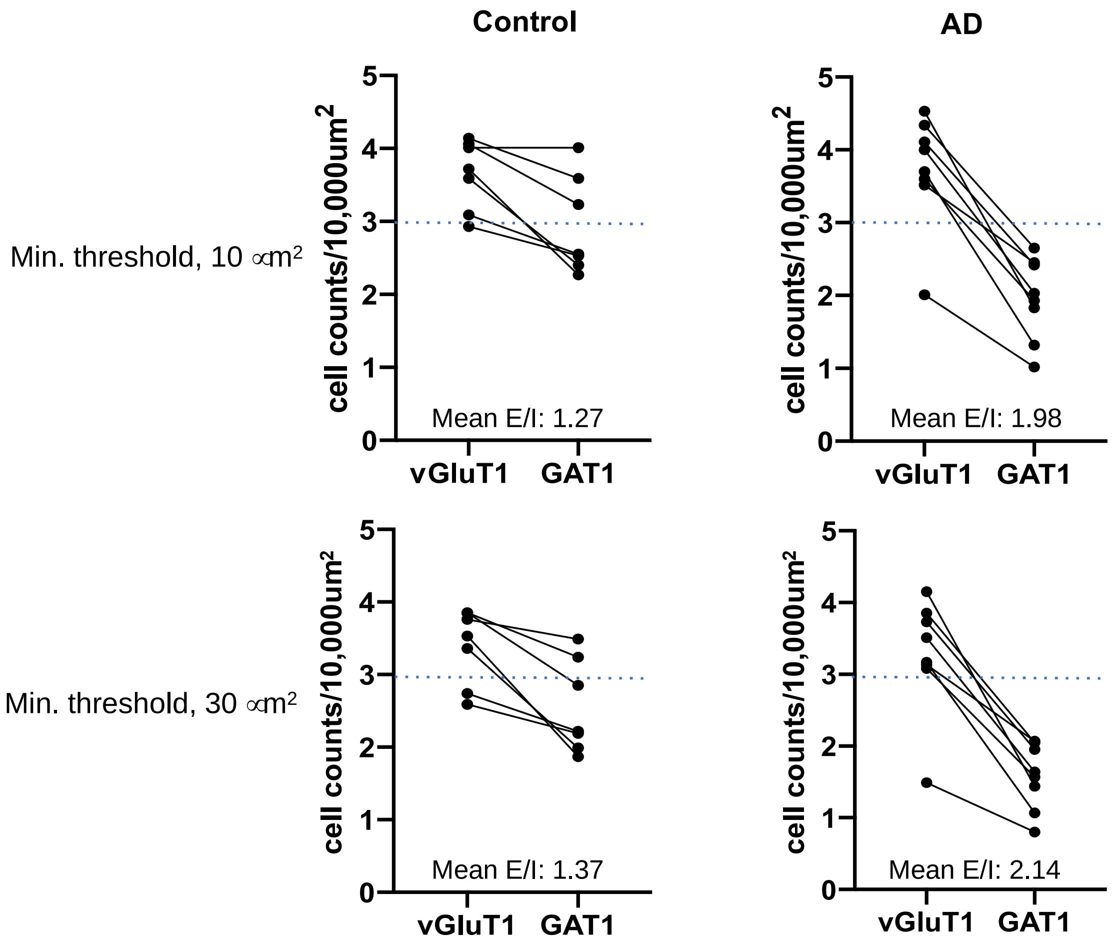


**Supplementary Fig 5**. Plots show the corresponding numbers of vGluT1 and GAT1 mRNA expressing cells per fixed area of parietal cortex for all cases. Top row shows data collected using a minimum threshold of 10 $\mu m^2$ for labeled profiles detected by the automated counter; this threshold was used for the final analyses and reported outcomes. Bottom row shows that increasing the minimum threshold size of labeled profiles to 30 $\mu m^2$ did not markedly change the overall differences between groups; the cellular E/I ratio was still significantly greater in the AD group versus the control. For comparison between groups in each row, an arbitrary reference point (dotted line) is shown. Mean E/I (vGluT1/GAT1) values for each group are present at bottom.

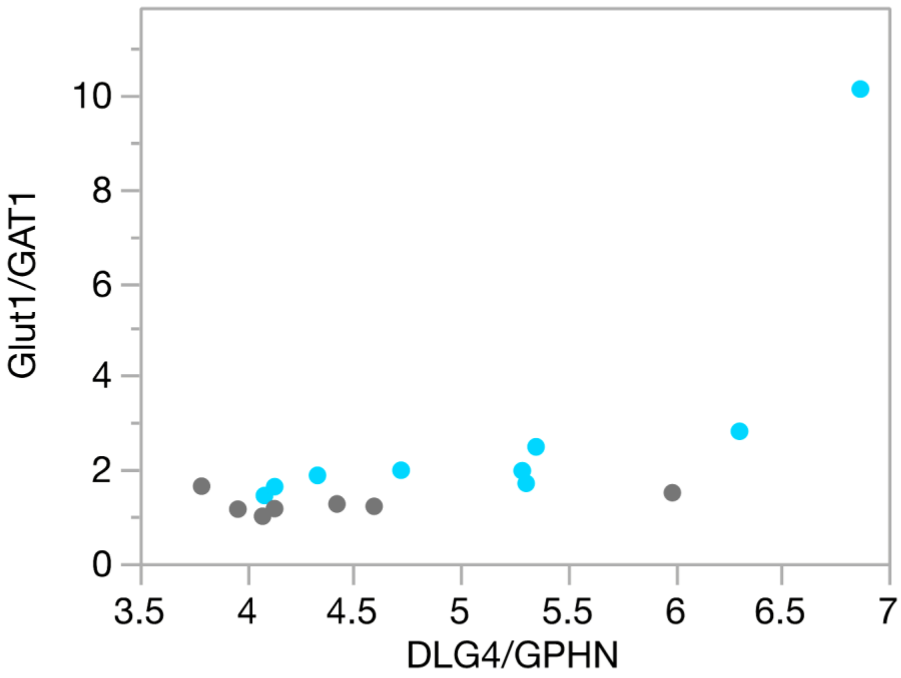


**Supplementary Fig 6**. Correlation between the cellular (vGluT1/GAT1) and transcriptional (DGL4/GPHN) expression E/I ratios for cases with both data sets, same as figure 7, but including subject H14.09.098 (see supplementary data 4 for cohort information) which was identified as an outlier by Mahalanobis distances UCL = 2.56. AD cases, cyan; control cases, black.
