# Supplementary material for: Increased excitatory to inhibitory synaptic ratio in parietal cortex samples from individuals with Alzheimer’s disease
Source: Nat Commun. 2021 May 10;12:2603. doi: 10.1038/s41467-021-22742-8 (PMC8110554; doi:10.1038/s41467-021-22742-8)
Supplement: Supplementary file 3 — Description of Additional Supplementary Files [file 41467_2021_22742_MOESM3_ESM.docx]

**Description of Additional Supplementary Files**

Supplementary Data 1.

Demographics of cohort for FDT and MSM studies.

Supplementary Data 2.

Summary electrophysiology and p-tau data.

Supplementary Data 3.

Correlations FDT, electrophysiology and p-tau data.

Supplementary Data 4.

Demographics of cohort for RNAseq and ISH studies.

Supplementary Data 5.

Genes positively correlated with the DLG4/GPHN ratio.

Supplementary Data 6.

Enrichment GO analysis of genes positively correlated with DLG4/GPHN ratio.

Supplementary Data 7.

List of predetermined excitatory and inhibitory GO modules.

Supplementary Data 8.

Differential expression of predetermined GO modules.

Supplementary Data 9.

Genes within WGCNA modules.

Supplementary Data 10.

Primers used for ISH in the ADTBI Allen Study

Supplementary Data 11.

Dataset
