# Supplementary material for: Increased excitatory to inhibitory synaptic ratio in parietal cortex samples from individuals with Alzheimer’s disease
Source: Nat Commun. 2021 May 10;12:2603. doi: 10.1038/s41467-021-22742-8 (PMC8110554; doi:10.1038/s41467-021-22742-8)
Supplement: Supplementary file 15 — Reporting Summary [file 41467_2021_22742_MOESM15_ESM.pdf]

nature research

Corresponding author(s): Agenor Limon, Julie Lauterborn

Last updated by author(s): Mar 3, 2021

Reporting Summary

Nature Research wishes to improve the reproducibility of the work that we publish. This form provides structure for consistency and transparency in reporting. For further information on Nature Research policies, see our Editorial Policies and the Editorial Policy Checklist.

Statistics

For all statistical analyses, confirm that the following items are present in the figure legend, table legend, main text, or Methods section.

n/a	Confirmed	
☐	☒	The exact sample size (*n*) for each experimental group/condition, given as a discrete number and unit of measurement
☐	☒	A statement on whether measurements were taken from distinct samples or whether the same sample was measured repeatedly
☐	☒	The statistical test(s) used AND whether they are one- or two-sided<br>*Only common tests should be described solely by name; describe more complex techniques in the Methods section.*
☐	☒	A description of all covariates tested
☐	☒	A description of any assumptions or corrections, such as tests of normality and adjustment for multiple comparisons
☐	☒	A full description of the statistical parameters including central tendency (e.g. means) or other basic estimates (e.g. regression coefficient) AND variation (e.g. standard deviation) or associated estimates of uncertainty (e.g. confidence intervals)
☐	☒	For null hypothesis testing, the test statistic (e.g. *F*, *t*, *r*) with confidence intervals, effect sizes, degrees of freedom and *P* value noted<br>*Give P values as exact values whenever suitable.*
☒	☐	For Bayesian analysis, information on the choice of priors and Markov chain Monte Carlo settings
☒	☐	For hierarchical and complex designs, identification of the appropriate level for tests and full reporting of outcomes
☐	☒	Estimates of effect sizes (e.g. Cohen's *d*, Pearson's *r*), indicating how they were calculated

Our web collection on statistics for biologists contains articles on many of the points above.

Software and code

Policy information about availability of computer code

Data collection	For the FDT analyses, we provide a description in the Methods that all images were collected and deconvoluved using Volocity 4.1 (Perkin Elmer). For flowcytometry data collection the Guava Soft 2.7® software was used. For recording of electrophysiological experiments Strathclyde Electrophysiology Software WinEDR V3.2.7 software was used.
Data analysis	For FDT analyses, we describe in the Methods that all images were analyzed using in-house software built using Matlab R2007, Perl v5.30.0, and C99 to quantify all labeled puncta within the size constraints of synapses. We have used this method in numerous (>10) publications from our group and citations are included in the manuscript.<br>Guava Soft 3.3® software was used to analyze flowcytometry data.<br>pCLAMP™ 11 was used to analyze electrophysiological data.<br>GraphPad Prism v.8 and JMP v.14 were used for statistical testing and plotting of the graphs.<br>WGCNA version 1.69, R version 4.0 and ENSEMBL Biomart release 100 were used for analyses of transcriptomic data modules<br>Scikit-image 0.16.2 and Phyton 3.7 were used for automatic count of cells in ISH analysis. The DOI for cell counter code is at: DOI: 10.5281/zenodo.4558112

For manuscripts utilizing custom algorithms or software that are central to the research but not yet described in published literature, software must be made available to editors and reviewers. We strongly encourage code deposition in a community repository (e.g. GitHub). See the Nature Research guidelines for submitting code & software for further information.

Data

Policy information about availability of data

All manuscripts must include a data availability statement. This statement should provide the following information, where applicable:

- Accession codes, unique identifiers, or web links for publicly available datasets
- A list of figures that have associated raw data
- A description of any restrictions on data availability

Source Data are provided with this paper in the “source data file” and Supplementary data 11. Transcriptomic and ISH data is publicly available from https://aging.brain-map.org. Specifically, normalized RNA seq data can be downloaded from: https://aging.brain-map.org/download/index, and ISH images can be downloaded by accessing case information: from https://aging.brain-map.org/donors/summary.

The following databases were used in this paper.
Allen Aging, Dementia and Traumatic Brain Injury study: https://aging.brain-map.org
Metascape, Gene Annotation and Analysis Resource: https://metascape.org/gp/index.html#/main/step1
BioMart (ENSEMBL version 100): http://www.biomart.org

Field-specific reporting

Please select the one below that is the best fit for your research. If you are not sure, read the appropriate sections before making your selection.

☒ Life sciences ☐ Behavioural & social sciences ☐ Ecological, evolutionary & environmental sciences

For a reference copy of the document with all sections, see nature.com/documents/nr-reporting-summary-flat.pdf

Life sciences study design

All studies must disclose on these points even when the disclosure is negative.

Sample size	The experimental design started with the analysis by FDT followed by downstream flow cytometry and MSM analyses of the same samples and adjacent tissue slices. Thus, the group size was determined by FDT and was selected on the basis of past experience and dictates of power analyses; power analysis determined that with the typical effect size and profile (e.g., change in of 20% ; sigma = 15; alpha = 0.05; power = 0.80) the minimal sample size to determine significance would be 5/group.
Data exclusions	For FDT, no data for objects identified by the automated system using criteria for synapse-size elements was excluded. For MSM oocytes were recorded within 18 to 36 hrs. For the estimation of the E/I ratio we only included oocytes were GABA and kainate currents had clear activation and deactivation phases, a signal to noise ratio of at least 3:1 and were consistently activated by multiple applications in the same oocyte.<br>For ISH analysis the subject H14.09.098 had poor staining signal for GAT1 which produced an artifactually large GluT1/GAT1 ratio, as evidenced by its identification as an outlier by Mahalanobis distances UCL=2.56 (Supplementary Fig. 5), thus this subject was not included in the analyses. The inclusion of this subject can be seen in supplementary figure 5.
Replication	The E/I imbalance results for AD brains were demonstrated by two independent approaches in one cohort (n = 16 cases total) using fluorescence deconvolution tomography (FDT) & microtransplantation of synaptic membranes (MSM); the complementary use of these approaches and the validation of the findings is a central point of the work. In addition, we further validated the main findings using publicly available RNA-Seq data from the Aging, Dementia, and TBI Study (ADTBI) (https://aging.brain-map.org) in an independent cohort (n= 20 cases). The data was further confirmed by in situ hybridization (ISH) in samples with this information from the ADTBI study.<br>For primary FDT and amyloid plaques analyses, all tissues were processed and analyzed together as described in Methods. Numerous images were taken from each case and independently analyzed; thus, results were based on 10-12 image stacks per brain for FDT, and 20 images per brain for plaques as stated in the Methods. Fow cytometry data results were confirmed in two independent analysis. MSM data findings were replicated by recordings performed in the University of California Irvine and the University of Texas Medical Branch. Gene expression analysis findings were confirmed by three different approaches; direct hypothesis-driven statistical test of difference in expression levels of specific genes, fold change expression in predetermined excitatory and inhibitory gene ontology modules and weighted gene co-expression analysis (WCGNA) to determine unbiased modules of gene co-expression. For the in situ hybridization analyses, the findings were replicated using two threshold sizes for the labelled cells and both outcomes are reported.
Randomization	Randomization was not necessary since the tissue samples came from post-mortem human subjects with specific diagnoses. The control, AD and DS groups in the cohort used for amyloid plaques, FDT, flow cytometry and MSM measurements, was balanced for the covariates of age and sex. The neuropathological change and daily living scale (mBALDS) were balanced in AD and DS. Postmortem intervals were longer in the control group, however, two independent studies show that the PMI does not affect the FDT (Lauterborn et al. Brain Pathol 30, 319-331 (2020)) or MSM measures (Scaduto P et al., Sci Rep 10, 8626 (2020). For gene expression and ISH no randomization was done because we used all available tissue in the dataset with the specific diagnosis to compare with our first cohort.
Blinding	For FDT and amyloid plaques count studies, all tissue samples from the human cases were numerically coded and processed for immunocytochemistry, photographed, and ran through analytical systems with the experimenters blind to condition. Initial organization and graphing of all output data also was done blind. The code was finally broken when data was organized by group, and for group statistics. For For flow cytometry and MSM analyses the experimenters were blind to diagnosis and the code was broken after initial group statistics. The

MSM experiments were done at UCIrvine and then at University of Texas Medical Branch by different people blind to the identity of the samples. For gene expression and ISH analysis data blinding was not possible as the data was obtained from publicly available already coded for diagnosis. However, the hypothesis testing was clearly defined a priori using all cases available and the dataset as described in Methods.

Reporting for specific materials, systems and methods

We require information from authors about some types of materials, experimental systems and methods used in many studies. Here, indicate whether each material, system or method listed is relevant to your study. If you are not sure if a list item applies to your research, read the appropriate section before selecting a response.

Materials & experimental systems

n/a	Involved in the study
☐	☒ Antibodies
☒	☐ Eukaryotic cell lines
☒	☐ Palaeontology and archaeology
☐	☒ Animals and other organisms
☒	☐ Human research participants
☒	☐ Clinical data
☒	☐ Dual use research of concern

Methods

n/a	Involved in the study
☒	☐ ChIP-seq
☐	☒ Flow cytometry
☒	☐ MRI-based neuroimaging

Antibodies

Antibodies used

mouse anti-PSD-95 (Thermo Scientific, #MA1-045), rabbit anti-GPHN (Abcam, #ab32206), Donkey anti-mouse Alexa Fluor 488 (Thermo Fisher Scientific, #A21202) and Donkey anti-rabbit Alexa Fluor 594 (Thermo Fisher Scientific, #A21207).

Validation

MA1-045 (clone 6G6-1C9; antibody registry RRID #AB-325399) is one of the most a widely-used monoclonal antibodies for PSD-95; The ThermoScientific website indicates that over 150 publications have used it, and it has been used and validated across many applications including immunocytochemistry (84 publications), immunofluorescence (34 publications) western blotting (71 publications), and immunoprecipitation (11 publications). It has reported species reactivity with human, mouse, and rat. The rabbit anti-GPHN ab32206 antibody (RRID# AB-2112628) has been used in 12 publications as indicated on the Abcam website including in human; it also has specificity for mouse, and rat. In addition, it has been tested and validated for immunocytochemistry and western blotting by the company.

Animals and other organisms

Policy information about studies involving animals; ARRIVE guidelines recommended for reporting animal research

Laboratory animals

Sexually mature females Xenopus laevis frogs to harvest oocytes

Wild animals

No wild animals were used in this study

Field-collected samples

No field collected samples were used in this study

Ethics oversight

All frogs were handled in accordance with the guidelines of the National Institute of Health guide for care and Use of Laboratory animals, and with the approval of the institutional Animal Care and Use Committee of the University of California Irvine (IACUC-1998-1388) and the University of Texas Medical Branch (IACUC-1803024).

Note that full information on the approval of the study protocol must also be provided in the manuscript.

Flow Cytometry

Plots

Confirm that:

☒ The axis labels state the marker and fluorochrome used (e.g. CD4-FITC).

☒ The axis scales are clearly visible. Include numbers along axes only for bottom left plot of group (a 'group' is an analysis of identical markers).

☒ All plots are contour plots with outliers or pseudocolor plots.

☒ A numerical value for number of cells or percentage (with statistics) is provided.

Methodology

Sample preparation

A description of the extraction of synaptosome-enriched P2 fractions is provided in Methods. P2 samples were diluted 1:4000

	in PBS before to perform flow cytometry assay.
Instrument	Guava® easyCyte Flow Cytometer
Software	Guava® InCyte™ software
Cell population abundance	On average, 34.1 (± 2.8) of the particles were included in the synaptosomes gate (1-3µm)
Gating strategy	The gating parameters were established using known size beads (Spherotech, Inc.) to exclude all the particles out of the 1 to 3 µm range that include typical synaptosomes as reported previously (Micci et al, 2019; PMID 31200742) . Further, three internal gates were built to separated small (1 µm < diameter of particle < 2 µm), medium (2 µm = diameter) or large (2 µm < diameter < 3 µm) synaptosomes-like particles using the beads as references.

☒ Tick this box to confirm that a figure exemplifying the gating strategy is provided in the Supplementary Information.
